# ACCEPTABILITY OF ANIMATRONIC PETS AMONG COMMUNITY-DWELLING VETERANS LIVING WITH DEMENTIA AND THEIR CAREGIVERS

**DOI:** 10.1093/geroni/igac059.2222

**Published:** 2022-12-20

**Authors:** Melissa Harris, Edwin Alcorn, Judith Davagnino

**Affiliations:** Duke University, Durham, North Carolina, United States; Durham VA Health Care System, Durham, North Carolina, United States; Durham VA Health Care System, Durham, North Carolina, United States

## Abstract

Animatronic pets can improve psychosocial outcomes among older adults, but little is known about in-home use by Veterans living with dementia and the perceptions of informal caregivers regarding these tools. This quality improvement project examined the usability and acceptability of animatronic pets by community dwelling Veterans living with dementia from the perspective of informal caregivers. Usability and acceptability were examined through a structured telephone interview with 17 caregivers. Usability was operationalized as frequency of: pet use by the Veteran (1=Not at all, 4=Daily or all of the time), and reminders provided by caregivers (1=Not at all, 4=Daily or all of the time). Acceptability was defined as perceived benefit (measured using an acceptability tool; 0= Not at all, 2=A great deal beneficial) and satisfaction (measured using acceptability tool with close and open-ended response items) as reported by caregivers. Results indicated high usability (71% of Veterans used the pet daily with reminders provided by 30% of caregivers) and acceptability (Benefit: (M=1.5/2, SD=0.7); Satisfaction: 94% of caregivers would continue to encourage use of the animatronic pet by the Veteran and recommend one to others). Open-ended responses suggest that they may be less satisfying to those who lost a real pet or did not have an affinity for pets earlier in life. Animatronic pets can be used with minimal assistance by Veterans with dementia and are acceptable to informal caregivers. As VA initiatives support provision of these pets, additional research is needed to examine their broader impact on Veterans living with dementia specifically.

